# Guidelines for Cancer Treatment during Pregnancy: Ethics-Related Content Evolution and Implications for Clinicians

**DOI:** 10.3390/cancers14174325

**Published:** 2022-09-03

**Authors:** Alma Linkeviciute, Rita Canario, Fedro Alessandro Peccatori, Kris Dierickx

**Affiliations:** 1 Legal Tech Center, Mykolas Romeris University, LT-08303 Vilnius, Lithuania; 2 Fertility and Procreation Unit, Division of Gynecologic Oncology, European Institute of Oncology, IRCCS, 20141 Milan, Italy; 3 Cancer Metastasis, i3S-Institute for Research & Innovation in Health, R. Alfredo Allen 208, 4200-135 Porto, Portugal; 4 Research Centre, Portuguese Oncology Institute of Porto, 4200-072 Porto, Portugal; 5 ICBAS, School of Medicine and Biomedical Sciences, R. Jorge de Viterbo Ferreira 228, 4050-313 Porto, Portugal; 6 Centre for Biomedical Ethics and Law, KU Leuven, 3000 Leuven, Belgium

**Keywords:** cancer treatment during pregnancy, clinical practice guidelines, biomedical ethics principles, oncology, breast cancer

## Abstract

**Simple Summary:**

Clinical practice guidelines for cancer treatment during pregnancy have been available for the past 20 years; however, whether they contain bioethical guidance is currently unknown. A systematic medical literature review was performed to identify the presence of biomedical ethics principles present in guidelines published until 2021. Most of the included guidelines (25 out of 32) refer to biomedical ethics principles such as respect for patient’s autonomy, beneficence and justice. Earlier guidelines stress the importance of patient wishes and choices in light of limited evidence and vast unknowns while balancing maternal and fetal wellbeing. More recent guidelines tend to focus on evidence-based data to balance favorable outcomes for pregnant patients and their fetuses with counselling support to help the patients and their support network understand the rationale behind available treatment options. However, ethics-related content in such guidelines is not presented in a structured manner, indicating the need for methodological upgrades. Therefore, a more structured approach is needed when addressing existing and potential ethical issues in clinical practice guidelines for cancer treatment during pregnancy.

**Abstract:**

(1) Background: Current scientific evidence suggests that most cancers, including breast cancer, can be treated during pregnancy without compromising maternal and fetal outcomes. This, however, raises questions regarding the ethical implications of clinical care. (2) Methods: Using a systematic literature search, 32 clinical practice guidelines for cancer treatment during pregnancy published between 2002 and 2021 were selected for analysis and 25 of them mentioned or made references to medical ethics when offering clinical management guidance for clinicians. (3) Results: Four bioethical themes were identified: respect for patient’s autonomy, balanced approach to maternal and fetal beneficence, protection of the vulnerable and justice in resource allocation. Most guidelines recommended informing the pregnant patient about available evidence-based treatment options, offering counselling and support in the process of decision making. The relational aspect of a pregnant patient’s autonomy was also recognized and endorsed in a significant number of available guidelines. (4) Conclusions: Recognition and support of a patient’s autonomy and its relational aspects should remain an integral part of future clinical practice guidelines. Nevertheless, a more structured approach is needed when addressing existing and potential ethical issues in clinical practice guidelines for cancer treatment during pregnancy.

## 1. Introduction

Pregnancy-related cancer is a relatively rare entity, affecting approximately 1 in 1000 pregnancies [1,2,3]. Nevertheless, as there is a consistent postponement of pregnancy in almost all middle- and high-income countries [4], incidence may increase. Breast cancer is the main oncological disease affecting reproductive age women worldwide and the most common type during pregnancy [5], followed by lymphoma, cervical cancer and thyroid cancer [3,6]. The clinical approach to these complex patients depends not only on the type and stage of the tumor, but also on the pregnancy trimester, and involves a personalized diagnostic and therapeutic approach [7], because cancer diagnosis and treatment during pregnancy pose unique challenges [6]. There is a vast amount of literature regarding cancer treatment during pregnancy that demonstrates that different cancer treatments can safely be conducted during pregnancy without compromising the fetal outcomes [8], namely, different chemotherapy agents, surgery and radiotherapy [9]. This, however, depends on the gestational age of the fetus at the time of treatment, tumor type and disease stage, as treatments may induce developmental teratogenicity [10,11,12]. There are a number of ethical questions involved, especially when oncological prognosis is dire and a patient’s desire for parenthood is strong; practical questions such as breastfeeding after breast cancer are also frequently triggered in this setting [13,14]. Nevertheless, knowledge regarding bioethical aspects arising during this unusual co-occurrence is scarce [15].

Clinical practice guidelines for cancer management in the course of pregnancy provide advice for healthcare professionals to assist their decision-making process throughout the course of their patients’ illness management [16]. However, whether the available guidelines offer guidance regarding the ethical aspects of cancer care during pregnancy and which type of bioethical content is present remains to be compiled. Therefore, the aim of this paper is to review and consolidate the medical ethics guidance provided in clinical practice guidelines for cancer treatment during pregnancy by identifying the core ethical concepts referenced in these documents, analyzing time-trends and demonstrating the implications of this guidance on clinical breast cancer and other cancers management during pregnancy.

This work builds on the foundations created by an earlier research project carried out at the European Institute of Oncology (Milan, Italy) looking at biomedical ethics concepts/principles present in clinical practice guidelines, which sought to understand the standpoints and approaches to ethical issues surrounding cancer care during pregnancy through clinical practice guidelines [16]. Whether the approaches and standpoints expressed in the clinical practice guidelines are ethical in themselves is beyond the scope of this work. Furthermore, the authors did not seek to evaluate the quality of the scientific evidence for cancer treatment during pregnancy presented in the analyzed guidelines.

## 2. Materials and Methods

A systematic review was conducted to identify clinical practice guidelines offering general or cancer-type specific management of oncological conditions in pregnant patients, adhering to standards laid out in the PRISMA (Preferred Reporting Items for Systematic Reviews and Meta-Analysis) statement [17]. The systematic review has been registered at Research Registry reviewregistry1442. Due to the nature of this research, it did not require Institutional Review Board (IRB) approval. The data search was conducted in two steps, consisting of an initial search of clinical practice guidelines up to 2015 [16], which was further updated in 2021. The search was performed using an electronic database (PubMed) and included articles published in the English language until 14 July 2021, with no retrospective time limit. Other databases were not searched based on previous experience, where Web of Science and Science Direct databases did not yield any additional results, nor did the snowballing approach, to those already identified through the PubMed search. Grey literature, citation track and hand-search were not conducted due to resource constraints and the low likelihood of identifying additional publications.

The following string of search terms was used (pregnancy AND cancer AND guideline) to identify the publications that oncologists and other healthcare professionals would possibly refer to when looking for practical guidance for pregnancy-related cancer care. Two eligibility criteria were established for including the clinical practice guidelines in this review: (1) guidelines should be published as articles and expert meeting reports aiming to provide recommendations for cancer treatment and management during pregnancy; (2) guidelines should be released, reviewed and/or endorsed by a professional society representing clinicians practicing in a clinical field related to oncology. Review articles where the primary goal was to provide guidelines for the management of tumors diagnosed during pregnancy but not related to any professional organization were excluded due to a potential opinion bias, based on the assumption that professional clinical organizations would present a consensual expert view.

The content of the guidelines was analyzed by looking for references to four biomedical ethics principles as defined by Beauchamp and Childress: respect for autonomy, nonmaleficence, beneficence and justice [18], and the European specification of basic ethical principles in bioethics and biolaw: autonomy, dignity, integrity and vulnerability [19]. The content analysis was conducted by the first author in consultation with the supervising authors, with whom difficult cases were discussed.

The selected guidelines were analyzed using the critical interpretative approach [20] by ascribing the wording in clinical practice guidelines to definitions used in medical ethics, since biomedical ethics principles are not referred to directly in the analyzed clinical practice guidelines. Table 1 shows what biomedical ethics principles were identified in the analyzed guidelines and Table 2 illustrates how original wording from the guidelines corresponded to medical ethics concepts. For example, “*attention to patient’s personal wishes*” [21], “*abortion is a personal decision*” [22] were interpreted as respect for patient’s autonomy, while “*considerations should be given to the health of both mother and foetus*” [23] was regarded as balancing maternal and fetal beneficence but “*maximizing maternal outcome*” [24] was assumed as a preference to maternal beneficence. “*Pregnancy should never be interrupted*” was regarded as protection of the vulnerable [25] and “*reasonable course of action based on current knowledge, available resources and the needs of the patient”* [26] was considered as a reference to the biomedical ethics principle of justice.

## 3. Results

**Figure 1:**
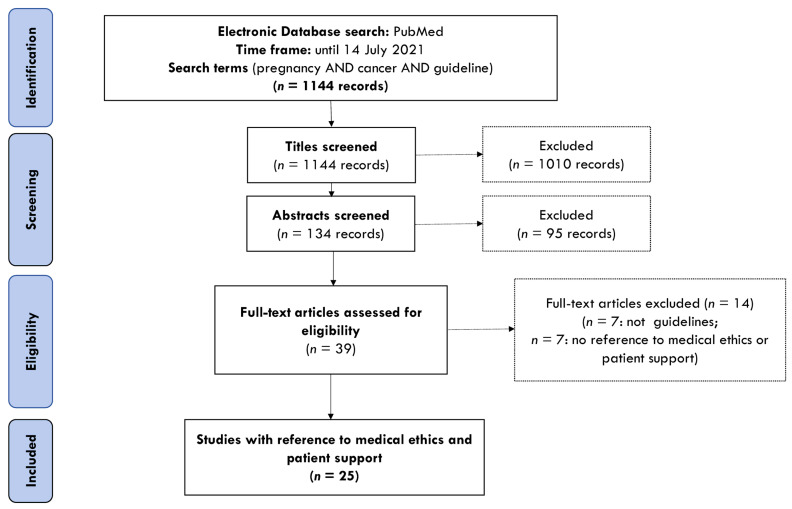
Study Flowchart.

A total of 32 guidelines were included for full-text analysis. From these, 7 were excluded due to the lack of references to patient care outside the clinical aspects and 25 guidelines were included in this systematic review, as detailed in Figure 1. Most of the guidelines referred to a site-specific cancer type (breast [27,28,29,30,31], cervix [32,33], thyroid [25,34,35], melanoma [26,36], leukemia [23,37] and Hodgkin lymphoma [21]) and the remaining ones to groups of pathologies [24,38,39,40] or women cancers and their treatment in general [22,41,42,43,44,45]. Regarding the quality of the included guidelines, it is important to stress that guidance in some professional guidelines was retrieved from case studies, case study reviews and various registries, whereas in others, guidance was based on expert opinion rather than on systematically collected data [16].

Four biomedical ethics concepts were identified in the reviewed guidelines, as summarized in Table 1 and Table 2. Autonomy, beneficence and vulnerability were transversal throughout the 20-year period. Consideration of justice in resource allocation only appeared in 2015. Respect for patients’ autonomy was the most ubiquitous and dominant ethical principle, as illustrated by the following statements: “*the implications of starting chemotherapy in the third trimester and the risks to the woman of delaying chemotherapy to gain advantage for the baby need to be discussed with the mother”* [23], *“personal priorities for each patient will clearly influence the decision”* [21], “*termination of pregnancy is an individual decision affected by many factors*” [22].

The beneficence principle was the second ethical concept identified, with guidance mentioning a balanced approach to maternal and fetal beneficence: *“Consideration should be given to the health of both mother and baby and the informed wishes of the mother. The woman should be fully informed about the diagnosis, treatment of the disease and possible complications during pregnancy*” [23], “*The priority must be the health of the mother*” [21] and “*serious consideration should be given to the option of treating breast cancer whilst continuing with the pregnancy*” [29]. Some guidelines also introduced concepts surrounding protection of the vulnerable, as exemplified with this sentence: “*State-of-the-art treatment should be provided for this vulnerable population to preserve maternal and fetal prognosis*” [24].

A relatively new theme emerging in clinical practice guidelines was reasonable resource allocation, considering a large picture of a healthcare system and available resources. This suggests that resources have to be considered based on current knowledge and the needs of the patient to deliver effective and safe medical care “*practitioner will follow a reasonable course of action based on current knowledge, available resources and the needs of the patient to deliver effective and safe medical care*” [26].

Overall, experts advocate for a multidisciplinary approach to cancer treatment during pregnancy, evidence-based medicine and counselling services for patients. Reference to concepts that can be framed within the ethical principles has changed during the 20-year period analyzed. Some guidelines published before 2015 recognized the importance of personal relationships (relational autonomy) that pregnant cancer patients might be inclined to consider when making their treatment choices: for example, partner and other children, if present: “*possibly involving her partner and family in the decision- making process*” [41], “*the pregnant woman, her family, and her medical team are required to make complex treatment decisions*” [44], “*discussion should allow appropriate time for reflection and should possibly involve the partner, if present*” [27]. All guidelines referring to breast cancer during pregnancy were published before 2015 [27,28,29,30,31] where more focus was given to a patient’s preferences since little evidence-based data was available at that time.

Later guidelines published after 2015 continue to stress the importance of supporting pregnant cancer patients to take informed decisions about their cancer treatment and pregnancy care. This is achieved by providing accessible information about available medical care options and their implications to a pregnant patient and their developing fetus [26,32,33,35,37,39]. For example, “*feeling informed and in control through the provision of information can lead to women feeling engaged and active in their treatment decisions leading to better patient outcomes*” [33], “*every patient must be counselled by a multidisciplinary team. This team should consist of experts in the fields of gynaecologic oncology, neonatology, obstetrics, anaesthesiology, radiation oncology, medical oncology, psychooncology, and, if requested, theology or ethics*” [32]. It also continues to emphasize the importance of respect for patient autonomy and taking a patient’s wishes into account when determining the disease management plan [32,33,35,37,39]. For example, “*each woman needs to make the decision that fits her best after an in-depth discussion; clinical care teams should be supportive of her choice, whatever that choice may be*” [37]. Some guidelines also recognize the importance of the relational aspect of respect for patient autonomy “*counselling should be offered to both the affected woman and her partner*” [40], “*women and their partners should be counselled that no guidelines exist regarding how best to monitor chronic myeloid leukaemia during pregnancy*” [37]. However, guidance appears to have shifted from focusing on patients’ personal wishes in the unknown circumstances to counselling, where treatment options are discussed in light of the available scientific evidence. The latter might not be well reflected in currently available guidelines for breast cancer treatment during pregnancy because these guidelines were released before consolidated scientific evidence became more widely available.

There has been one guideline identified which encourages protecting the fetus while ensuring supporting care for its mother “Pregnancy should never be interrupted. Women with differentiated thyroid carcinoma (DTC) and no evidence of aggressive or advanced disease may be reassured that most DTC are slow growing and that surgery soon after delivery is unlikely to change prognosis” [25]. Subsequent guidelines from the past six years do not refer to the protection of the vulnerable, which was seen in the guidelines published earlier. In the earlier guidelines, protection of the vulnerable was defined broadly, including pregnant women, cancer patients, unborn children/fetuses, neonates and children [24,29].

**Table 1:** Main biomedical ethics concepts/principles identified in the guidelines.

Principles	Expressions of the Principle	Guidelines Mentioning This Principle
**Autonomy**		
Enabling patients to take informed decisions	Providing patients with the information about available treatment options, including risks and benefits for the mother and the fetus	[22,23,26,27,29,30,31,32,33,34,35,36,37,38,39]
Respect for patient’s autonomy	Involving the patient in a decision-making process by informing her about the options and taking patient’s wishes into account when determining the disease management plan.	[21,22,23,27,28,32,33,34,35,36,37,38,39,43]
Acknowledging the respect for relational autonomy	Involving the patient and her partner/family in a decision-making process by informing about the options and taking patient wishes into account when determining the disease management plan.	[22,28,30,37,38,40,41,42,43,44]
**Beneficence**		
Balancing maternal and fetal beneficence	Considering maternal health outcomes and fetal risks when determining the disease management plan.	[23,26,29,32,33,35,37,38,40,45]
Maternal beneficence	Giving preference to maternal health outcomes over fetal risks, if optimal balance for both is not possible.	[21,24,26,44]
Fetal beneficence	Giving more weight for protecting the fetus and allowing pregnancy to continue but offering support for the pregnant patient.	[25]
**Vulnerability**		
Protection of the vulnerable	Proving care and support for those who might be under-represented or not able to defend their position. Could include pregnant women, cancer patients, unborn children/fetuses, neonates, children.	[24,29]
**Justice**		
Reasonable resource allocation	Following a reasonable course of action based on current knowledge, available resources and the needs of the patient to deliver the effective and safe medical care.	[26]
**No biomedical ethics principles/concepts referenced**		
No references to patient care outside clinical aspects	Focuses on providing clinical guidance without making suggestions how patient care should be handled in a light of ethics.	[46,47,48,49,50,51,52]

**Table 2:** Review summary and key findings of clinical practice guidelines addressing cancer management during pregnancy.

Reference; Organization	Guideline Description	Management Recommendation	Patient Support	Biomedical Ethics Concepts/Principles Referenced in the Guideline
**Reed et al., 2021** [33]; British Gynaecological Cancer Society (BGCS)	Attempts to cover management of invasive ***cervical cancer*** reflecting diagnosis and imaging including new imaging and sentinel lymph node biopsies.	Multidisciplinary team, evidence-based medicine	Feeling informed and in control through the provision of information can lead to women feeling engaged and active in their treatment decisions leading to better patient outcomes.	*Balancing maternal and fetal beneficence, autonomy*
**Amant et al., 2019** [40]; International Network on Cancer, Infertility and Pregnancy (INCIP), third international consensus meeting	Promotes effective management of pregnant women with ***gynecological cancers*** and their offspring.	Multidisciplinary team, evidence-based medicine	Pregnant cancer patients deserve a careful continuous assessment and support of their psychological wellbeing on a routine basis with follow-up in the postpartum period. Counselling should be offered to both the affected woman and her partner.	*Balancing maternal and fetal beneficence, relational autonomy*
**Cibula et al., 2018** [32]; The European Society of Gynaecological Oncology (ESGO), the European Society for Radiotherapy and Oncology (ESTRO), and the European Society of Pathology (ESP)	Provides clinically relevant and evidence-based guidelines in order to improve the quality of care for women with ***cervical cancer*** across Europe and worldwide	Primary aims of recommended treatment plan are oncological safety of the pregnant woman, as well as survival without additional morbidity of the fetus.	Every patient diagnosed with CCIP must be counselled by a multidisciplinary team. This team should consist of experts in the fields of gynecologic oncology, neonatology, obstetrics, anesthesiology, radiation oncology, medical oncology, psycho-oncology, and, if requested, theology or ethics.	Multidisciplinary team recommends an individual consensual treatment plan according to patient’s intention, tumor stage, and gestational age of pregnancy at cancer diagnosis (*respect to patients’ autonomy, balancing maternal and fetal beneficence*)
**Coccia et al., 2018** [45]; National Comprehensive Cancer Network	Focuses on treatment and management considerations for Adolescent and Young Adult (AYA) patients with cancer.	Women diagnosed with cancer during pregnancy require individualized treatment from a multidisciplinary team involving medical, surgical, and radiation oncologists, gynecologic oncologists, obstetricians, and perinatologists as appropriate. In addition to the disease characteristics in pregnant women, the gestational age of the fetus is a significant factor in the selection of treatment.	Referral to tertiary cancer centers with expertise in the diagnosis of cancer during pregnancy and maternal–fetal medicine and knowledge of the physiologic changes that occur during pregnancy should be strongly encouraged. Offer psychosocial support and counselling to help alleviate distress.	The goals of controlling maternal cancer and providing the fetus the best chance for survival with normal development (*treatment recommendations suggest balancing maternal and fetal beneficence*)
**Alexander et al., 2017** [35]; American Thyroid Association (ATA)	Informs clinicians, patients, researchers, and health policy makers on published evidence relating to the diagnosis and management of ***thyroid disease*** in women during pregnancy, preconception, and the postpartum period. Offers strength of evidence to support recommendations.	In all women of childbearing age who are thyrotoxic, the possibility of future pregnancy should be discussed. Women with GD seeking future pregnancy should be counselled regarding the complexity of disease management during future gestation, including the association of birth defects with ATD use. ATD dose should be reduced to protect the fetus.	Preconception counselling should review the risks and benefits of all treatment options and the patient’s desired timeline to conception.	A careful balance is required between making a definitive diagnosis and instituting treatment while avoiding interventions that may adversely impact the mother, the health of the fetus, or the maintenance of the pregnancy. Surgery should be performed in the second trimester in order to minimize complications to both the mother and fetus. (*balancing maternal and fetal beneficence*)
**Pallera et al., 2017** [37]; National Comprehensive Cancer Network (NCCN)	Provides recommendations for the management of chronic-phase and advanced-phase ***Chronic Myeloid Leukemia (CML)*** in adult patients.	Clinical care teams should be prepared to address issues relating to fertility and pregnancy, as well as counsel these patients about the potential risks and benefits of treatment discontinuation and possible resumption of tyrosine kinase inhibitor (TKI) therapy should CML recur during pregnancy.	Before attempting pregnancy, women and their partners should be counselled that no guidelines exist regarding how best to monitor CML during pregnancy, nor how best to manage progressive disease should it occur during pregnancy. Conception while on active TKI therapy is strongly discouraged because of the risk of fetal abnormalities.	Each woman needs to make the decision that fits her best after an in-depth discussion regarding relapse rates off TKI therapy and treatment if needed during pregnancy, and clinical care teams should be supportive of her choice, whatever that choice may be; The potential risk/benefit balance should be carefully evaluated in terms of maternal health and fetal risk before initiation of treatment during pregnancy, especially during the first trimester. (*respect for patient’s autonomy; balancing maternal and fetal beneficence*)
**Lishner et al., 2016** [39]; International Consensus Meeting of Prenatal Hematologic Malignancies	Provides guidelines for clinical management of ***hematologic cancers*** during the perinatal period, which were developed by a multidisciplinary team including an experienced hematologist/oncologist, a high-risk obstetrics specialist, a neonatologist, and experienced nurses, social workers, and psychologists.	A multidisciplinary team—including, at minimum, an experienced hematologist/oncologist, a high-risk obstetrics specialist, a neonatologist, as well as experienced nurses, social workers, and psychologists, providing close follow-up—is critical to ensuring optimal maternal and fetal outcomes; Diagnosis followed by appropriate staging is essential and should not be delayed due to pregnancy; An overarching goal in the care of all pregnant patients with non-Hodgkin lymphoma (NHL) is delivery at term.	An informed decision to treat needs to be made with the patient, using dosimetry analyses provided by the medical physicist.	The decision to administer antenatal therapy is based on several factors, such as type of non-Hodgkin lymphoma (NHL), gestational age, and patient preference; Pregnancy termination recommended in early pregnancy if aggressive treatment is needed. (*balancing maternal and fetal beneficence*)
**Bluemel et al., 2015** [26]; written and approved by the European Association of Nuclear Medicine (EANM), discussed by distinguished experts from the EANM Oncology Committee, national nuclear medicine societies, the European Society of Surgical Oncology (ESSO) and the European Association for Research and Treatment of Cancer (EORTC) melanoma group. The document has been endorsed by the Society of Nuclear Medicine and Molecular Imaging (SNMMI).	Offers practice guidelines for nuclear medicine practitioners to help providing high-quality lymphatic mapping for the care of ***melanoma*** patients.	Management by multidisciplinary team; Risks and benefits of the procedures should be carefully discussed with the gynecologist; Radiolocalization of sentinel lymph nodes (SLNs) in patients with melanoma is associated with low levels of radiation exposure. While lymphatic mapping is not contraindicated in pregnant patients, it is common to halve the dose activity and same-day surgery is preferred.	The resources and facilities available for patient care may vary from one country to another and from one medical institution to another. Pregnant patients may be offered sentinel lymph node biopsy (SLNB) after careful counselling regarding the safety and efficacy of the procedure.	Practitioner will follow a reasonable course of action based on current knowledge, available resources and the needs of the patient to deliver the effective and safe medical care. (*respect for patient’s autonomy; balancing maternal and fetal beneficence; justice*)
**Ali et al., 2015** [23]; UK-based medical practitioners with expertise in acute myeloid leukaemia (AML), reviewed by Haemato-oncology Task Force of the British Committee for Standards in Haematology (BCSH), British Society of Haematology (BSH), UK AML National Cancer Research Network (NCRN)	Offers a uniformed consensus which, due to scarcity of the literature, is mainly based on expert opinion than trials for treating ***AML*** patients. Includes grade of evidence for all clinical recommendations	Management by multidisciplinary team; Pregnant patients with AML should be treated without delay after full and frank discussion; Beyond 32 weeks gestation it might be reasonable to deliver the fetus before chemotherapy.	The women should be fully informed about the diagnosis, treatment of the disease and possible complications; Reasons for and against elective termination should be discussed with a patient; Offer parents reassurance on baby’s health by performing tests and follow-up during infancy.	Considerations should be given to the health of both mother and fetus and informed wishes of the patient (*enabling patients to take informed decisions, respect for patient’s autonomy; balancing maternal and fetal beneficence*)
**Follows et al., 2014** [21]; UK-based medical experts and patients’ representatives; revised by Haemato-oncology Task Force of the British Committee for Standards in Haematology (BCSH)	The guideline for first line management of classical ***Hodgkin lymphoma***	The priority must be the health of the mother and, ideally, management should be in conjunction with an obstetrician experienced in high-risk pregnancies	Patient’s personal priorities should be taken into consideration when making treatment decisions	Attention to patient’s personal wishes (*respect for patient’s autonomy*); Maternal health and wellbeing should prevail (*maternal beneficence*)
**Amant et al., 2014** [24]; European Society of Gynecological Oncology (ESGO) task force “Cancer in Pregnancy” in concert with other international experts	Provides timely and effective guidance for pregnant women and health care providers to optimize maternal treatment and fetal protection and to promote effective management of the mother, fetus, and neonate when administering potentially teratogenic medications in ***gynecological malignancies***	To maximize the maternal outcome, cancer treatment should follow a standard treatment protocol as for non-pregnant patients; Despite limited evidence-based information, cancer treatment during pregnancy can succeed; Iatrogenic prematurity should be avoided; State-of-the-art treatment should be provided for this vulnerable population to preserve maternal and fetal prognosis.	Individualization of the treatment and effective psychological support is imperative to provide throughout the pregnancy period	Maximizing maternal outcome (*maternal beneficence*) Best treatment for vulnerable population [pregnant women with cancer] (*protection of the vulnerable*)
**Peccatori et al., 2013** [41]; European Society of Medical Oncology (ESMO), endorsed by Japanese Society of medical Oncology (JSMO)	Provides Clinical Practice Guidelines for managing patients diagnosed with cancer during pregnancy and provide guidance on fertility considerations for women desiring pregnancy following cancer diagnosis (***breast, cervical, lung cancer, leukemia*** and other tumors)	Referral to institution with expertise; Multidisciplinary team; Standard chemo might not be feasible in all cases; Target full-term delivery whenever possible; Pregnancy termination strongly discouraged	Involving a partner and family in decision-making process; Multidisciplinary care and counselling	Partner and family involvement in decision-making (*respect for relational autonomy*)
**Koren et al., 2013** [44]; Chemotherapy During Pregnancy Working Group, approved by the Society of Obstetricians and Gynaecologists of Canada (SOGC)	Reflects clinical and scientific advances and offers recommendations concerning ***chemotherapy use in pregnant women*** and women of child-bearing age	It is important to balance maternal and fetal risks; Decisions should be made individually for each patient; Multi-disciplinary team, including physicians and social workers, psychologists, spiritual advisors	Discuss the available options with pregnant patient and her family	Partner and family involvement in decision-making (*respect for relational autonomy*); Maternal health and wellbeing should prevail (*maternal beneficence*)
**Cardoso et al., 2012** [27]; European Society of Breast Cancer Specialists (EUSOMA)	Position paper, recommendations for treating young women with ***breast cancer***	Pregnancy after breast cancer should not in principle be discouraged	Issues of body image, sexuality, fertility and lactation must be discussed with young women with breast cancer; Counselling for family planning and contraception; Involve the partner if present	Informing the patient about treatment effects and family planning (*enabling patients to take informed decisions; respect for patient’s autonomy*); Partner involvement in decision-making (*respect for relational autonomy*)
**Dauer et al., 2012** [22]; Society of Interventional Radiology and the Cardiovascular and Interventional Radiology Society of Europe, endorsed by the Canadian Interventional Radiology Association	Intends to assist interventionalists and their staff in managing and counselling pregnant patients who need ***fluoroscopically or CT-guided interventional*** procedures	Interventions should be justified with the aim for doing more good than harm; Concern about the possible side effects of ionizing radiation exposure on the conceptus [fetus] should not preclude medically indicated diagnostic or interventional x-ray procedures when the medical benefit for the mother is justifiable; Conceptus doses lower than 100 mGy should not be considered a reason for terminating a pregnancy	Pregnant patients should be counselled based on sound information about the risks of radiation exposure; If possible pre and post procedure counselling should take place involving the mother and the father	Provide counselling support to patients (*enabling patients to take informed decisions*); Abortion is an individual decision affected by many factors (*respect for patient’s autonomy*); Partner involvement (*respect for relational autonomy* [*indirect reference*])
**De Groot et al., 2012** [34]; Endocrine Society Clinical Practice Guideline (US), reviewed and commented on by members of The Endocrine Society, Asia and Oceania Thyroid Association, and the Latin American Thyroid Society—Section 5. Thyroid Nodules and Cancer	Updates the guidelines for the management of ***thyroid dysfunction*** during pregnancy and postpartum published previously	No clear evidence that pregnancy worsens the survival of pregnant patient	Information for the patient making the decision about breastfeeding	Provide information for the patient (*enabling patients to take informed decisions; respect for patient’s autonomy* [*indirect reference*])
**Royal College of Ob/Gyn, 2011** [28]; Royal College of Obstetricians & Gynaecologists	Provides clinical guidance to health professionals caring for women of childbearing age with a diagnosis or history of ***breast cancer***. Green-top guideline No. 12	Suggests auditing the referrals and outcomes	People with cancer should be fully informed of potential gonadotoxicity before treatment, and specialist psychological support and counselling should be available; Involve a partner in a discussion with a multidisciplinary team	Informing the patient about treatment effects (*respect for patient’s autonomy*); Partner and family involvement in decision-making (*respect for relational autonomy*)
**Amant et al., 2010** [29]; an international expert Panel	Provides guidance for clinicians about the diagnosis, staging and treatment of ***breast cancer*** occurring during an otherwise uncomplicated pregnancy	Serious consideration should be given to continuing of pregnancy whilst treating cancer; Delivery should not be induced before 37 weeks as morbidity mainly associated with prematurity; Treatment should adhere as closely as possible to standard protocols; Breastfeeding shortly after chemotherapy not recommended	Multidisciplinary team should provide patient with clear explanation of treatment options	Seriously consider continuing of pregnancy whilst treating cancer (*balancing maternal and fetal beneficence, protection of the vulnerable* [*indirect reference*]); Provide information for the patient (*enabling patients to take informed decisions*)
**Marsden et al., 2010** [36]; endorsed or had an input from U.K. Melanoma Study Group, the British Association of Dermatologists, the British Association of Plastic, Reconstructive and Aesthetic Surgeons, the Royal College of Physicians, London, the Association of Cancer Physicians, the Royal College of Radiologists, London, the Royal College of Surgeons of England, the Royal College of Pathologists (pathology section only), the Royal College of General Practitioners, London, and the Department of Health	Guideline for management of ***cutaneous melanoma*** presents evidence-based guidance for treatment, with identification of the strength of evidence available at the time of preparation of the guidelines, and a brief overview of epidemiology, diagnosis, investigation and follow up.	Chemotherapy does not have survival benefit in IV stage; Melanoma can metastasize to the placenta and fetus more frequently which has poor prognosis for the mother and the baby	Social and family effects of developing recurrent melanoma during pregnancy or after birth are great. Counselling recommended.	Provide support, information and education to patients (*enabling patients to take informed decisions; respect for patient’s autonomy*)
**Papini et al., 2010** [25]; Joint statement for clinical practice by Italian associations on thyroid cancer	Provides guidance to thyroid nodule and differentiated ***thyroid cancer*** management in pregnancy	Most tumors are slow growing and surgery after the delivery will not change the prognosis; Pregnancy should never be interrupted	Assurance about prognosis should be given to the affected patients	Never interrupting the pregnancy (*fetal beneficence but also care and support for the expectant mother*)
**Pentheroudakis et al., 2010** [42]; European Society of Medical Oncology (ESMO)	Provides a guide with scientific levels of evidence for management of ***breast and cervical cancers, and melanoma***	The optimal therapeutic strategy should be jointly chosen by the medical team, patient and family and will depend on gestational age, nature and stage of cancer, treatment options and patient wishes	All patients at risk of infertility who have not completed childbearing should discuss germ-line storage options with a medical team	Partner and family involvement in decision-making (*respect for relational autonomy*)
**Amant et al., 2009** [38]; international experts in the field	Suggests models for treatment of ***gynecologic cancer*** in pregnancy	Randomized trials and prospective studies on cancer treatment during pregnancy are lacking; Multidisciplinary expertise should be available; It is advisable to engage the expertise of other members of the healthcare team such as psychologists, social and pastoral workers	Counselling both parents on the maternal prognosis and fetal risk is needed	The parents should be informed about the different treatment options and the possible consequences for the patient and the fetus (*enabling patients to take informed decisions; respect for relational autonomy* [*indirect reference*]); The prognosis, treatment modalities, gestational age, and patients’ preference are pivotal in the decision making process on treatment during pregnancy or termination of pregnancy (*balancing maternal and fetal beneficence* [*indirect reference*]; *respect for patient’s autonomy*)
**Pentheroudakis et al., 2008** [43]; European society of Medical Oncology (ESMO)	Provides recommendations for ***diagnosis, treatment and follow-up*** surrounding cancer treatment, fertility preservation and cancer during pregnancy	Pregnancy termination is advised in the case of chemotherapy or radiotherapy administration during the first trimester, need for radical gynecologic surgery, poor maternal life expectancy	The optimal therapeutic strategy should be jointly chosen by the medical team, patient and family and will depend on gestational age, nature and stage of cancer, treatment options and patient wishes.	Inclusion of patient and family in decision-making (*respect for relational autonomy* [*indirect reference*]); considering patient’s wishes (*respect for patient’s autonomy*)
**Loibl et al., 2006** [30]; Internal expert meeting	Offers guidelines on how to diagnose and treat women with ***breast carcinoma during pregnancy***	Multidisciplinary approach is recommended including psychologist, social workers, and a chaplain; Ongoing psychological support during treatment and delivery should be available for the patient and her family	A supportive patient-physician relationship is required, as is close collaboration and feedback of all disciplines involved in the patient’s care, aiming to assist the patient and her partner towards achieving a true informed consent and commitment to treatment	Informing the patient about the options (*enabling patients to take informed decisions*); involving partner in consultations (*respect for relational autonomy*)
**Helewa et al., 2002** [31]; Breast Disease Committee and Executive Committee and Council, Society of Obstetricians and Gynaecologists of Canada	Provides physicians with up-to-date, accurate information and recommendations regarding pregnancy and lactation impact on cancer risk, prognosis, risk of reoccurrence and feasibility of breastfeeding in women affected by ***breast cancer***; and also offers counselling recommendations	Multidisciplinary approach should be taken; Patients should be counselled regarding the effect of proposed on the fetus and on overall maternal prognosis; Termination of pregnancy should be discussed, but patient should be counselled that prognosis is not altered by termination of pregnancy	Counselling support for breast cancer patients is advocated	Informing the patient about the options (*enabling patients to take informed decisions*)

## 4. Discussion

This systematic review has summarized the key ethical concepts—based on the core biomedical ethics principles as defined by Beauchamp and Childress [18] and the European specification of basic ethical principles in bioethics and biolaw [19]—present in clinical practice guidelines of pregnancy-related cancer. We found that most of the guidelines assessed in this study (25 out of 32) contained ethical guidance, namely regarding autonomy, beneficence, vulnerability and justice, which were transversal throughout the 20-year period. The first two themes are found in classical biomedical ethics studies and are known as *moral principles* in modern biomedical ethics [18], while the third theme is considered a European bioethics principle [19], which is also known as a *principle of protection of the vulnerable* [53] and a *principle of respect for human vulnerability* [54]. The fourth theme was mentioned only in one guideline emphasizing the importance of the reasonable use of available resources [26].

Globally, there was a predominance of guidance regarding respect for patients’ autonomy and beneficence (balanced approach for mother and fetus). Due to reassuring evidence that maternal cancer can be treated effectively without compromising the fetal outcomes, there has been a growing number of guidelines supporting the balancing of maternal and fetal beneficence [26,32,33,35,37,40,45], which is especially evident in the guidelines released in the last six years (2015–2021). For example, “*the potential risk/benefit balance should be carefully evaluated in terms of maternal health and foetal risk before initiation of treatment during pregnancy*” [37], while maternal health outcomes are only prioritized if an optimal balance for a pregnant patient and their fetus is not possible “*should be carefully discussed with the gynaecologist considering the risks and benefits”* [26] and if aggressive disease is detected in the early stages of the pregnancy [39].

A time-trend was observed in the guidelines published in the last quarter analyzed (2016–2021), which focus on counselling the patients (and often their partners) about informed treatment decisions, which may reflect the growing availability of safety data [1,3]. Nevertheless, as more robust medical findings become available for cancer treatment during pregnancy, there is a risk of shifting clinical practice towards the medical side of evidence-based balanced beneficence while reducing the focus on respect for patients’ wishes and choices, without considering other variables. Recognition and support of a patient’s autonomy and its relational aspects should remain an integral part of future clinical practice guidelines for cancer treatment during pregnancy. This will be an important aspect to consider when updated clinical practice guidelines are being released for breast cancer management during pregnancy, because currently available guidelines for this cancer type that include ethical guidance date back to 2012–2013 [27,41].

It is widely recognized that having pregnant cancer patients as active participants in their care and treatment planning is crucial for good maternal and fetal clinical outcomes [33]. This indicates that pregnant patients are being considered as active participants in clinical decision making about available treatment options which seek the best evidence-based outcomes for them and their developing fetuses. The focus on the patient (also known as patient centricity) is seen across most guidelines which corresponds with mainstream healthcare practice, where patient centric efforts have continuously been made to make healthcare delivery more patient focused through patient empowerment, personalized care and relational/care ethics. Patient centricity can be considered as putting the patient first in an open and sustained engagement with the attending clinical team, who respectfully and compassionately work together with the patient to achieve the best care experience and clinical outcome for that person and their family [55]. Overall, the analyzed clinical practice guidelines can be considered patient-centric because treatment decisions are made together with the patient by providing evidence-based information and allowing time and space to consider the available options. It was evident in the included guidelines that patient counselling and decisional support is widely recognized as a standard of care for pregnant cancer patients [22,24,26,27,28,30,31,32,33,35,36,37,38,40,41,45]. Moreover, the patient is not left alone to make difficult moral choices and the most recent guidelines seem to support the maternalistic approach from the clinical teams by emphasizing the importance of available scientific evidence in patient counselling [14]. The content in clinical practice guidelines appears to support the ideas expressed elsewhere that clinicians cannot determine how the patients should view their disease, but when equipped with empathy and compassion clinicians can support their patients by explaining the logical rationale behind evidence-based clinical advice [13]. It also somewhat allows healthcare professionals to have an active, collaborative role in decision making. Furthermore, the resource allocation theme corresponding to the biomedical ethics principle of justice [18], which takes into consideration equity and utility of the chosen treatment plan, emerged in 2015. It is not consistently mentioned in other guidelines, but its importance has been noted in the literature [13] and it would be reasonable to expect that more future guidelines will include resource allocation and treatment futility in their ethical guidance.

This work has reviewed and consolidated the ethical content presented in pregnancy-related cancer clinical practice guidelines and analyzed time-trends for the first time. Nevertheless, this review has inherent limitations that need to be considered when analyzing the results. Firstly, the search and main analysis was conducted by the first author independently, which might have increased the likelihood of selection, coding and interpretation bias. Nonetheless, selection choices and analysis were discussed with the rest of the research team, attempting to overcome these limitations. Secondly, the search was limited to English language and the grey literature was not searched, which might have excluded guidance from some medical organizations that do not have their guidance indexed in PubMed. However, the authors believe that most of the clinically relevant organizations tend to publish in journals indexed in this database and therefore were included. Thirdly, the quality of scientific evidence presented in the selected guidelines was not evaluated systematically. Indeed, the primary interest was to identify the references to ethics while recognizing that such guidelines do not explicitly aim to provide ethics guidance and, therefore, evaluating the quality of the guidance was not in the scope of this research. Hence, further research and debate on how clinical practice guidelines can and/or should address ethical issues related to this difficult clinical area should continue. The need for actionable ethical guidance in clinical practice guidelines has been shown in other clinical areas as well [56].

## 5. Conclusions

This systematic review has compiled, for the first time, the ethical guidance present in clinical practice guidelines referring to pregnancy-related cancer. Although the majority of analyzed guidelines mentioned some biomedical ethics principles, it is important to stress that 7 out of 32 screened articles (22%) did not make any mention of ethical aspects relating to cancer during pregnancy care, including some very recent guidelines released between 2019 and 2021. Moreover, among the guidelines that mentioned ethical themes, the approach to ethical issues has not been structured or consistent. These data highlight the need for a structured approach when addressing existing and potential ethical issues in clinical practice guidelines for cancer management during pregnancy, as it would help healthcare professionals to provide high-quality, patient-centered care and be prepared to address ethical issues and concerns in their clinics proactively and professionally. Moreover, this review emphasizes the importance of practical ethics and humanities training for healthcare professionals to equip them with skills for ethically challenging clinical situations and of patient-centric training to ensure that patients are active participants in their care. Clinical guidelines methodology would also benefit from more diverse and inclusive input, such as the inclusion of patient advocates, bioethicists and other humanities scholars. Overall, this work underscores the need for more research regarding ethics in the clinical care of pregnant cancer patients and a more systematic inclusion of ethics themes in clinical practice guidance, which should encourage individual healthcare professionals to be more mindful of ethical issues in their practice and skilled in addressing and resolving ethical concerns in their clinics.
